# Bacteremia with an Unusual Pathogen:* Mycobacterium neoaurum*

**DOI:** 10.1155/2016/5167874

**Published:** 2016-10-11

**Authors:** Hesham Awadh, Munthir Mansour, Mahmoud Shorman

**Affiliations:** ^1^Department of Internal Medicine, MUSOM, Huntington, WV, USA; ^2^Department of Infectious Diseases, MUSOM, Huntington, WV, USA

## Abstract

*Mycobacterium neoaurum (M. neoaurum)* is an infrequently encountered cause of infection in humans. It is a member of the rapidly growing mycobacteria family. It predominately afflicts those with a compromised immune status and a chronically indwelling vascular access. Isolation of this organism is challenging yet the advent of 16s ribosomal sequencing paved the way for more sensitive detection. No treatment guidelines are available and treatment largely depends on the experience of the treating physician and nature of the isolate. We report a case of* M. neoaurum* bacteremia in an immune competent host, with a chronically placed peripherally inserted central catheter (PICC line).

## 1. Introduction

*Mycobacterium neoaurum* is a pigmented, rapidly growing member of the* Mycobacterium parafortuitum* complex which typically grows in less than one week. It was first isolated by Tsukamura and Mizuno in 1972 [1]. It can be found on environmental surfaces, soil in particular [2]. It is an extremely rare cause of infection in humans and has been infrequently reported as a cause of infection in literature. Most of the infections associated with this organism are associated with indwelling vascular catheters in the setting of an immunocompromised host. In this report we are presenting a case of an immune competent patient with a chronically indwelling vascular access (PICC).

## 2. Case Report

Our patient is a 67-year-old female patient with past medical history significant for diabetes mellitus complicated by chronic kidney disease, recurrent urinary tract infections with the background of chronically indwelling urinary catheter due to debility, and a chronically placed peripherally inserted central catheter (PICC) (for over six months) due to having a difficult peripheral intravenous access. She initially presented to a rural facility with picture of urinary tract infection. Blood and urine cultures were drawn and the patient was sent home on oral ciprofloxacin 500 mg once daily. After six days the patient was recalled for admission because her blood cultures started to grow acid fast bacilli, and she was sent to our facility for escalation of care. Vital signs on arrival showed a blood pressure of 98/58 mm/hg, heart rate of 88 beat/minute, respiratory rate 16/minute, and oxygen saturation 95% on ambient air. Physical examination was remarkable for a bed-bound, chronically-ill looking frail lady, not in apparent distress. Initial labs showed a white blood cell count of 5.5 × 10^9^/L, with 68.5% neutrophils, 21.1% lymphocytes, 2.1% monocytes, 2.9% eosinophils, ESR 112 mm/hour, CRP 2.7 mg/L, and serum lactic acid of 1.28 mg/L. Serum chemistry and an electrolyte panel as well as a liver function panel showed no significant anomalies apart from evidence of acute kidney injury. Urine microscopy was still suggestive of urinary tract infection being loaded with WBCs and bacteria. Blood and urine cultures were redrawn (central and peripheral) and were still pending at this point. Initial hypotension responded to saline boluses and the patient was admitted to the general medical ward. The patient was placed empirically on cefepime 1000 mg IV Q12 hours to treat the urinary tract infection. No antimycobacterial agents were started awaiting further identification of the acid fast bacilli. The PICC was removed on admission and the tip was sent for culture. At day 3 of hospitalization, urine cultures grew extended-spectrum *β*-lactamase* Escherichia coli*, and thus cefepime was stopped and the patient was started on ertapenem. At day 5 of hospitalization, branching acid fast bacilli were isolated from blood cultures as well as cultures from PICC tip culture.* Actinomyces* panel was negative and the blood cultures were sent to Mayo Clinic labs for further identification. Repeat blood cultures (after removal of PICC line) were declared negative and thus no antimycobacterial agents were started since the patient was stable, and ertapenem was continued. Later on,* Mycobacterium neoaurum* was identified at Mayo Clinic labs. The patient was started on ciprofloxacin and doxycycline as empirical therapy (sensitivity panel was not done), with the plan to be treated for at least 4 weeks. The patient lost follow-up after discharge.

## 3. Discussion

*Mycobacterium neoaurum* is a rapidly growing, scotochromogenic* Mycobacterium* of the* parafortuitum* complex (group 4 according to the Runyon classification) [3]. It is abundant in nature, present in soil, dust, rocks, and water. It has the ability to survive harsh environmental conditions in that it has been isolated in extremes of temperature and milieu with very low PH [2]. This species is extremely hydrophobic and has a unique ability to form biofilms, which in addition to being an important survival mechanism makes it harder to isolate and eradicate. It also explains the affinity to develop device related infections. Other risk factors include immunosuppression, multiple comorbid conditions, and prior antibiotic therapy [2].

The first reported case of human infection with this organism was reported in 1987 in an elderly lady afflicted by ovarian cystadenocarcinoma metastatic to the peritoneum [4]. She had a chronically indwelling Hickman catheter previously inserted for parenteral nutrition.* Mycobacterium neoaurum* can result in an interestingly wide range of pathologies in spite of its rarity. Its ability to form biofilms make the majority of cases associated with foreign devices as stated above [2]. Examples include Hickman catheters, Broviac catheters, PICC lines [5], and arteriovenous fistula that included a polytetrafluoroethylene graft [6], pace makers [7], and prosthetic valve endocarditis [8]. Non-device related infections have also been reported: the first pulmonary infection was reported in 2006, and it was theorized that chronic inhaled corticosteroid therapy predisposed to this infection [9]. The first central nervous system (CNS) infection was reported when 16s rDNA was used to identify* Mycobacterium neoaurum* in brain tissue samples in an elderly female with rapidly progressive dementia [10], which was surrounded by controversy [11].

Isolation of this organism depends on initial detection via routine aerobic blood cultures (BacT/Alert; bioMérieux), determination of acid fast status using conventional methods, and high pressure liquid chromatography for identification at species level [12]. The advent of 16S ribosomal ribonucleic acid (16S rRNA) sequencing has become the golden standard in identification of the organism [13].

No treatment guidelines are available, and treatment depends on the sensitivity results and the experience of the treating physician. Multiple regimens were used with good response. Device removal was employed in the majority of the device-associated cases with rare exceptions. In a retrospective study that was conducted at The University of Texas MD Anderson Cancer Center published in 2012 about outcome of treatment of rapidly growing mycobacteria in cancer patients [14] it was concluded that device removal in addition to treatment with an antibiotic combination of at least two agents is usually associated with an excellent response. Antimicrobials used included macrolides, quinolones, aminoglycosides, carbapenems, and tetracyclines. Duration of treatment is not specified but treatment for 4–6 weeks has been reported [14, 15].

In our case, the patient was not immunosuppressed but at the same time had multiple comorbid conditions (chronic kidney disease, diabetes mellitus, hypertension, hyperlipidemia, and advanced osteoarthritis) in addition to being bed bound and debilitated. She had a chronically indwelling PICC and urinary catheter. The patient also had a history of multiple hospitalizations for multiple reasons and has a history of exposure to multiple antibiotic regimens. Initial step in the management was removal of PICC which was followed by resolution of the bacteremia. She was started on ertapenem for treatment of the urinary tract infection (in addition to catheter exchange), with improvement in clinical condition. The pathogenic role of* Mycobacterium neoaurum* in this clinical setting is not clear due to the presence of concomitant urinary tract infection, but we theorize that PICC removal in addition to initial treatment with ertapenem resulted in improvement of the bacteremia as well. No antimycobacterial agents were started until the result of the cultures were confirmed in Mayo Clinic labs, which warranted starting ciprofloxacin and doxycycline for 4 weeks. The patient was discharged in astable condition. The patient lost follow-up after discharge.

## 4. Conclusion

Device related infections are an important emerging cause of morbidity. Infections with* Mycobacterium neoaurum* are rare and often occur in immunocompromised patients. They can sometimes present with undifferentiated fever. Proper antibiotic therapy and device removal are usually associated with an excellent response.
